# Incisional hernia and its impact on health-related quality of life after cytoreductive surgery and hyperthermic intraperitoneal chemotherapy: a national prospective cohort study

**DOI:** 10.1186/s12957-018-1382-x

**Published:** 2018-04-25

**Authors:** Sissel Ravn, Henriette Vind Thaysen, Sanne Harsløf, Mette Møller Sørensen, Lene Hjerrild Iversen

**Affiliations:** Department of Surgery, Colorectal Surgical Unit, Aarhus University Hospital, Tage-Hansens Gade 2, DK-8000 Aarhus C, Denmark

## Abstract

**Background:**

To evaluate the incidence of incisional hernia (IH) after cytoreductive surgery and hyperthermic intraperitoneal chemotherapy (CRS + HIPEC) and its impact on health-related quality of life (HRQoL).

**Method:**

From June 2006 until June 2016, 152 patients were followed after CRS + HIPEC at Aarhus University Hospital, a single national center. Patients were seen postoperatively in an outpatient clinic at 3, 6, 12, 18, 24, 36 48, and 60 months. Clinical examinations at these follow-up visits were used to evaluate IH events prospectively. The incidence of IH was estimated using competing risk analysis and is presented as the cumulative incidence proportion (CIP). We expected the incidence to be 15% at 12 months. HRQoL was assessed at 12 months by the Short Form (SF-36) questionnaire, which we used to compare patients with an IH to patients without an IH.

**Results:**

The median follow-up time was 16.6 months [range 0.9–62.0]. During this period, 14/152 (9.2%) patients developed an IH. The 1-year CIP was 5.9% [95% CI 2.9; 10.4] (*n* = 8), and the 2-year CIP was 9.2% [95% CI 5.3; 14.5] (*n* = 14). Patients with an IH were significantly older (67 years [range 48–72]) compared to patients without IH (60 years [range 24–75], *p* ≤ 0.01). The rate of postoperative complications between patients with and without IH was comparable, except that a greater proportion of patients with IH had a fascial dehiscence (21.4%) compared to patients without an IH (3.6%). Reponses to the SF-36 show that patients with an IH report lower HRQoL with regard to *Role-physical* (mean difference − 32.9 [95% CI − 60.6; − 5.3]) and *Role-emotional* (mean difference − 20.2 [95% CI − 43.4; 3.1]), meaning a reduction in work and daily activities due to their physical and psychological health. We found no general decrease in HRQoL.

**Conclusion:**

CRS + HIPEC do not increase the risk of IH as measured within 12 months postoperatively, contrary to expectations. However, patients with an IH report a limitation in daily activities, which can best be explained by changes in physical and psychological health. A larger cohort from multiple centres is necessary to verify our findings.

## Background

Peritoneal carcinomatosis (PC) originating from colorectal cancer has conventionally been considered a terminal condition with a median life expectancy of 5–7 months [1]. Now, limited PC is managed, with selected patients, by using cytoreductive surgery (CRS) followed by hyperthermic intraperitoneal chemotherapy (HIPEC); this combination is an extensive procedure performed as a laparotomy [2]. CRS + HIPEC is associated with substantial postoperative morbidity [3], but beneficial long-term survival rates have been described [4].

An incisional hernia (IH), a common late complication following laparotomy, has been reported with a variable incidence throughout the literature [5]. A systematic review of 14,618 patients who underwent a midline incision reported the rate of IH to be 12.8% [range 0–35.6] and reported the time of presentation to be at a weighted mean of 23.7 months [6]. Approximately 50% of IHs develop within the first postoperative year; 90% develop within 5 years [7]. Investigating IHs, long-term follow-up is essential, and at least 3 years is recommended [8, 9].

Approximately 60% of IHs are asymptomatic, but a variety of symptoms, including conditions requiring emergency surgery, have been reported [10]. Several risk factors for developing IHs have been described: a previous IH, open surgery, placement of incision, closing technique, wound infection and dehiscence, and obesity [6, 11]. Additionally, smoking and use of preoperative steroids and cytostatic drugs/chemotherapy have been suggested to increase the risk of IHs [12]. This causality has been suggested to be due to its association with a delayed healing process of the abdominal wound [13].

The presence of IHs are known to negatively affect health-related quality of life (HRQoL) and perceptions of body image [14]. HRQoL is an especially important consideration when survival is gained at the expense of major surgical procedures or toxicity [15].

The hypothesis that CRS + HIPEC reduces the abdominal wall healing and increases the risk of IH has been suggested by Boutros et al., who conducted an experimental trial with prophylactic mesh placement in eight patients undergoing CRS + HIPEC, and within the sparse mean follow-up of 6.3 months, only one patient required re-laparotomy and presented subsequently with an IH [16]. However, no previous studies have investigated the incidence of IH following CRS + HIPEC. Since this extensive surgery is combined with administration of intraoperative local chemotherapy, we hypothesized that CRS + HIPEC is associated with an increased risk of developing a postoperative IH, compared with other laparotomy procedures. We expected the incidence to be 15% at 12 months. Secondly, we investigated the impact of IHs on HRQoL.

## Methods

### Patients

This study was carried out as a national observational prospective cohort study. In total, 160 patients who underwent CRS + HIPEC at the Department of Surgery, Aarhus University Hospital (AUH), in the period from June 2006 to April 2015 were included. Eight patients were excluded because they presented with an IH involving the midline before their CRS+HIPEC was performed, leaving 152 (95%) patients eligible for participation. These patients were followed until July 2016.

### Eligibility criteria

Patients with PC from colorectal cancer, appendix cancer, and patients with pseudomyxoma peritonei, and malignant peritoneal mesothelioma were eligible for CRS+HIPEC International standard criteria for CRS + HIPEC were followed, and the PC extent was estimated by the Dutch Seven Region Count Score (Dutch score), since this was the prognostic score applied by the surgical department from the beginning [4, 17].

### Preoperative evaluation

Patients were evaluated by contrast-enhanced (positron emission tomography (PET))-computed tomography (CT) of the thorax, abdomen, and pelvis and discussed at a multidisciplinary team conference, i.e., according to international standards [4]. Some patients received a preoperative diagnostic laparoscopy.

### CRS with HIPEC

CRS + HIPEC was initiated with an incision from xiphisternum to the pubic symphysis. A re-evaluation of possible contraindications was performed at the commencement of surgery. Three experienced consultant surgeons performed all peritonectomy procedures, with curative intent. While the abdomen was still open, a 90-min HIPEC perfusion with mitomycin C was performed. The midline incision was sutured continuously with a monofilament suture. Perioperative intravenous antibiotic prophylaxis with cefuroxime and metronidazole were given for 3 days.

### Postoperative management

To assess any complications, patients were scheduled for at least 1 day of intensive care, followed by at least 14 days of hospitalization. All patients, except patients with pseudomyxoma peritonei with low-grade neoplasia, were offered postoperative systemic adjuvant chemotherapy for 3–6 months.

### Follow-up

Follow-up visits were scheduled for 3, 6, 12, and 18 months and 2, 3, 4, and 5 years after the CRS + HIPEC. Each visit included a clinical examination supplied by a contrast-enhanced (PET)-CT of the thorax, abdomen, and pelvis.

### Outcome measures

The primary endpoint was the development of an IH, defined as any postoperative herniation at the vertical midline laparotomy incision. All events were evaluated and diagnosed at follow-up by a clinical examination performed by the same three surgeons who executed CRS + HIPEC. The date of IH development was recorded as the date it was diagnosed during a clinical examination. Any repair of the IH was identified through a review of medical records.

The secondary endpoint was the impact of an IH on HRQoL 12 months after CRS + HIPEC. HRQoL was compared between patients with and those without an IH.

### HRQoL assessment

At each follow-up, HRQoL was assessed by handing out three questionnaires, unless patients were informed of cancer recurrence. All three questionnaires were validated in Danish versions: the European Organization for Research and Treatment of Cancer (EORTC) Quality of Life Core Questionnaire (QLQ-C30) (version 3.0) [18]; the EORTC Quality of Life Colorectal Questionnaire module (QLQ-CR38) [19]; and the Short Form (SF-36) (acute version) [20, 21]. The questionnaires were administered at the 12-month follow-up visit, even though the exact time since surgery could vary by a few months. No preoperative HRQoL was assessed.

The primary questionnaire, SF-36, consists of generic 36 questions related to the patient’s general health [22]. The EORTC QLQ-C30 and the EORTC QLQ-CR38 are cancer- and colorectal-specific questionnaires, including 30 and 38 questions, respectively, that target functional and symptomatic aspects of HRQoL, including one global health status scale. The raw scores from all three questionnaires were aggregated into a linear score ranging from 0 to 100. A high score on the function and health scale indicates a good health, whereas a high score on the symptom scale indicates a high burden of symptoms [23]. A score was calculated if the patient had answered at least half of the items on the scale [24].

### Selection of scales for HRQoL

HRQoL variations between two groups—patients with IH (+IH) and patients without IH (−IH)—were compared. Specific scales from the questionnaires were selected on the basis of those with most relevance to IHs. From the SF-36, the scales related to physical functioning, role-physical, bodily pain, social functioning, and role-emotional were selected, while the scales from EORTC QLQ-C30 were role-, emotional-, and social-functioning, fatigue, pain, nausea/vomiting, constipation, diarrhea, and global health status. Finally, the selected endpoints from EORTC QLQ-CR38 were body image, symptoms from the gastrointestinal tract, and future perspective scales.

### Statistical analysis

Categorical variables are presented as numbers and percentages, while continuous variables are presented as medians with ranges. Equality between groups (patients ±IH) was tested using a chi-squared test for categorical variables and a Mann-Whitney *U* test for continuous variables. IH incidence was estimated using a competitive risk function and presented as the cumulative incidence proportion (CIP). The date of IH was considered a failure. Competing risks were considered as following events, whichever came first: end of the 5-year follow-up period, the most recent date for follow-up at our department, recurrence (of PC or development of distant metastasis), death from any cause, or June 2016. Date of recurrence was defined as the date when the patient was informed of recurrence, either detected by (PET)-CT, biopsy, or at surgery.

Regarding HRQoL, scales are presented as the average mean scores with standard deviations. Due to ceiling and floor effects in the HRQoL data, equality of mean scores between groups are presented as the difference of mean along with 95% confidence intervals [24]. However, differences of 10 points or more in average HRQoL scores were considered to show a clinically relevant difference, which should allow for the detection of moderate changes in HRQoL [25]. The level of statistical significance was 5%. The statistical analysis was performed using STATA statistical software (STATA, release IC14, STATACorp LP, Texas, USA).

## Results

### Patient and treatment characteristics

All 152 patients undergoing CRS + HIPEC in the period from June 2006 to April 2015 were followed. Overall, the median follow-up was 16.6 months [range 0.9–62.0]. During this period, 14/152 (9.2%) patients developed an IH. As expected, the median follow-up was lower for +IH patients compared to −IH patients (+IH 8.8 months [range 1.6–25.6] vs. −IH 18.3 months [range 0.9–62.0]), due to end of follow-up in patients with an IH diagnosis.

The baseline characteristics of patients are summarized in Table 1. Equality of means of +IH patients and −IH patients is presented.

**Table 1:** Patient Characteristics. Characteristics and postoperative course of patients with and without incisional hernia after cytoreductive surgery (CRS) with hyperthermic intraperitoneal chemotherapy(HIPEC)

Characteristics	*Patients with incisional hernia* *N = 14*	*Patients without incisional hernia* *N = 138*	*Total* *N = 152*	*P-value*
Sex				0.47
Male	6 (42.9)	46 (33.3)	52 (34.2)	
Female	8 (57.1)	92 (66.7)	100 (65.8)	
Age in years (median, range)	67 (48-72)	60 (24-75)	60 (24-75)	<0.01
ASA score (median, range)	2 (1-2)	2 (1-2)	2 (1-2)	0.11
Body Mass Index (median, range)	25.9 (21.3-32.0) *n=10	24.9 (17.2-39.2) *n=110	25.0 (17.2-39.2) *n=120	0.66
Preoperative neoadjuvant chemotherapy	9 (64.3)	66 (48.5) *n= 136	75 (50)	0.26
Origin of peritoneal carcinomatosis				0.93
Pseudomyxoma peritonei	5 (35.7)	39 (28.3)	44 (29.0)	
Colorectal cancer	5 (35.7)	63 (45.7)	68 (44.7)	
Appendix cancer	3 (21.4)	30 (21.7)	33 (21.7)	
Malignant peritoneal mesothelioma	1 (7.2)	6 (4.3)	7 (4.6)	
Number of involved regions of peritoneal carcinomatosis at time of CRS (No.) (median, range)	3 (1-7)	3 (0-7)	3 (0-7)	0.75
Duration of surgery (h) (median, range)	5.9 (4.0-8.0)	5.5 (1.7-16.7)	5.6 (1.7-16.7)	0.70
Postoperative complications during hospital stay	5 (35.7)	53 (38.4)	58 (38.2)	0.84
Surgical complications (events, %)**				
Fascial dehiscence	3 (21.4)	5 (3.6)	8 (5.3)	
Early fascia dehiscence ≤30 days postoperatively requiring re-operation	1 (33.3)	3 (60.0)	4 (50.0)	
Late fascia dehiscence >30 days postoperatively				
Treated conservatively	1 (33.3)	0 (0.0)	1 (12.5)	
Requiring re-operation	1 (33.3)	2 (40.0)	3 (37.5)	
Surgical site infection	0 (0.0)	0 (0.0)	0 (0.0)	
Intraabdominal abscess requiring drainage	1 (7.1)	3 (2.2)	4 (2.6)	
Anastomotic leak	0 (0.0)	2 (1.4)	2 (1.3)	
Intestinal fistula	0 (0.0)	1 (0.7)	1 (0.7)	
Pelvic abscess spontaneous emptied through vaginal stump	0 (0.0)	1 (0.7)	1 (0.7)	
Blow out of rectal stump	0 (0.0)	1 (0.7)	1 (0.7)	
Medical complications (events, %)**				
Febrilia e causa	2 (14.3)	13 (9.4)	15 (9.9)	
Pneumonia	1 (7.1)	13 (9.4)	14 (9.2)	
Pleura effusion requiring drainage	1 (7.1)	12 (8.7)	13 (8.6)	
Urinary tract infection	1 (7.1)	9 (6.5)	10 (6.6)	
Other complications***	1 (7.1)	20 (14.5)	21 (13.8)	
30-day mortality	0 (0.0)	1 (0.7)	1 (0.7)	0.75
Hospital stay at the department (days) (median, range)	15 (13-70)	14 (10-44)	14 (10-70)	0.18
Referred to local hospital for further recreation	4 (28.6)	44 (31.9)	48 (31.6)	0.80
Received postoperative adjuvant chemotherapy	2 (14.3)	47 (34.1)	49 (32.2)	0.64

*Number of patients

**: Number of events are too few allowing a statistical analysis

***Other complications included: Surgical: Pancreatic fistula, bladder tamponade (in the same patient with pancreatic fistula), diaphragm rupture (after diaphragm resection), hepatic haematoma (in the same patient with diaphragm rupture), postoperative bleeding requiring re-operation, rupture of the stomach (in the patient with postoperative bleeding), bleeding pyloric ulceration, leakage from aberrant biliary tract, haemothorax treated with streptase. Medical: Lung embolus (in the same patient with pancreatic fistula and bladder tamponade), oliguria because of dehydration, atrio-ventricular blockage requiring pacemaker, transient atrial fibrillation, exacerbation of known psychiatric disease, urinary retention requiring suprapubic drainage (history with prostatism), respiratory insufficiency, atrial fibrillation requiring medical treatment

+IH patients were significantly older (67 years [range 48–72]) compared to −IH patients (60 years [range 24–75]).

The rate of postoperative complications for +IH patients and −IH patients was comparable, except that a greater proportion of +IH patients had a fascial dehiscence compared to −IH patients (21.4% (+IH) vs. 3.6% (−IH)). None of the 152 patients developed a surgical site infection (SSI) in the postoperative period.

### Incisional hernias

**Fig. 1:**
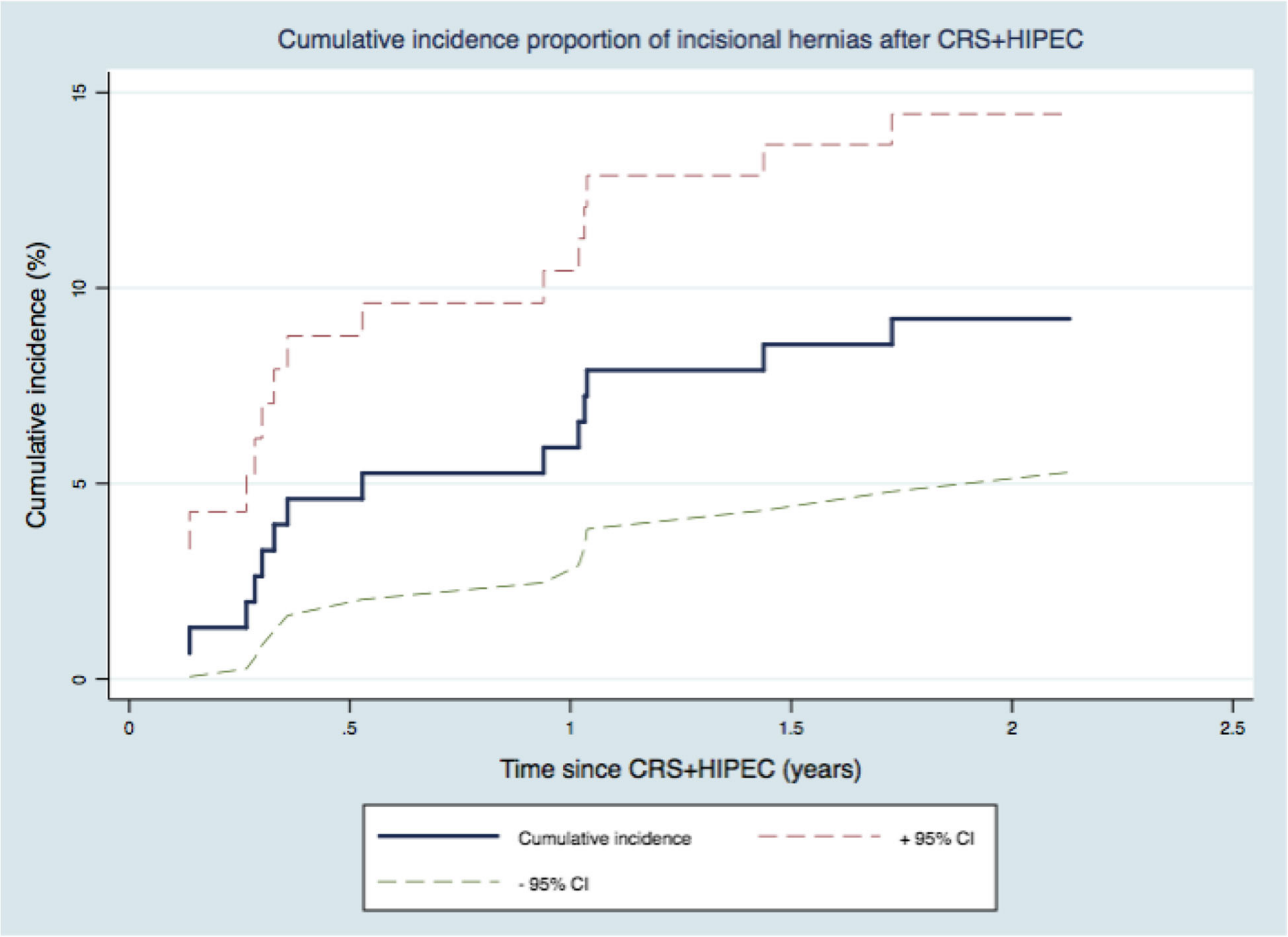
The cumulative incidence proportion of incisional hernias in 152 patients after cytoreductive surgery and hyperthermic intraperitoneal chemotherapy

Cumulatively, 5.9% [95% CI 2.9; 10.4] (*n* = 8) developed IHs within the first year, and 9.2% [95% CI 5.3; 14.5] (*n* = 14) developed IHs within 2 years, respectively. The CIP is presented graphically in Fig. 1.

In total, 4/14 (28.6%) + IH patients underwent hernia repair within the follow-up period. According to descriptions in the medical records, the indications for repair were acute operation due to obstructive ileus (*n* = 1); repeating events of sub-ileus and pain (*n* = 1); the feeling of severe heaviness (*n* = 1); and no description (*n* = 1). Repair was discussed, but assessed as clinically irrelevant, in 2/14 (14.3%) cases.

### HRQoL

Regarding HRQoL responses, a total of 73/103 (70.9%) patients answered the three questionnaires during their 12-month follow-up visit. Among these, 9/73 (12.3%) had presented with an IH prior to completing the questionnaires (data not shown). The response rate can be explained as follows: among the original 152 patients, two had passed away and 12 had a recurrence prior to the 12-month follow-up, leaving 138 patients who were scheduled for the12-month follow-up examination. Among these, two patients were followed by phone only and did not appear for a physical examination at 12 months. A further 33 patients had previously been informed of the recurrence of their cancer and were consequently not handed out a questionnaire. This left 103 patients who were considered eligible to receive all three questionnaires.

**Fig. 2:**
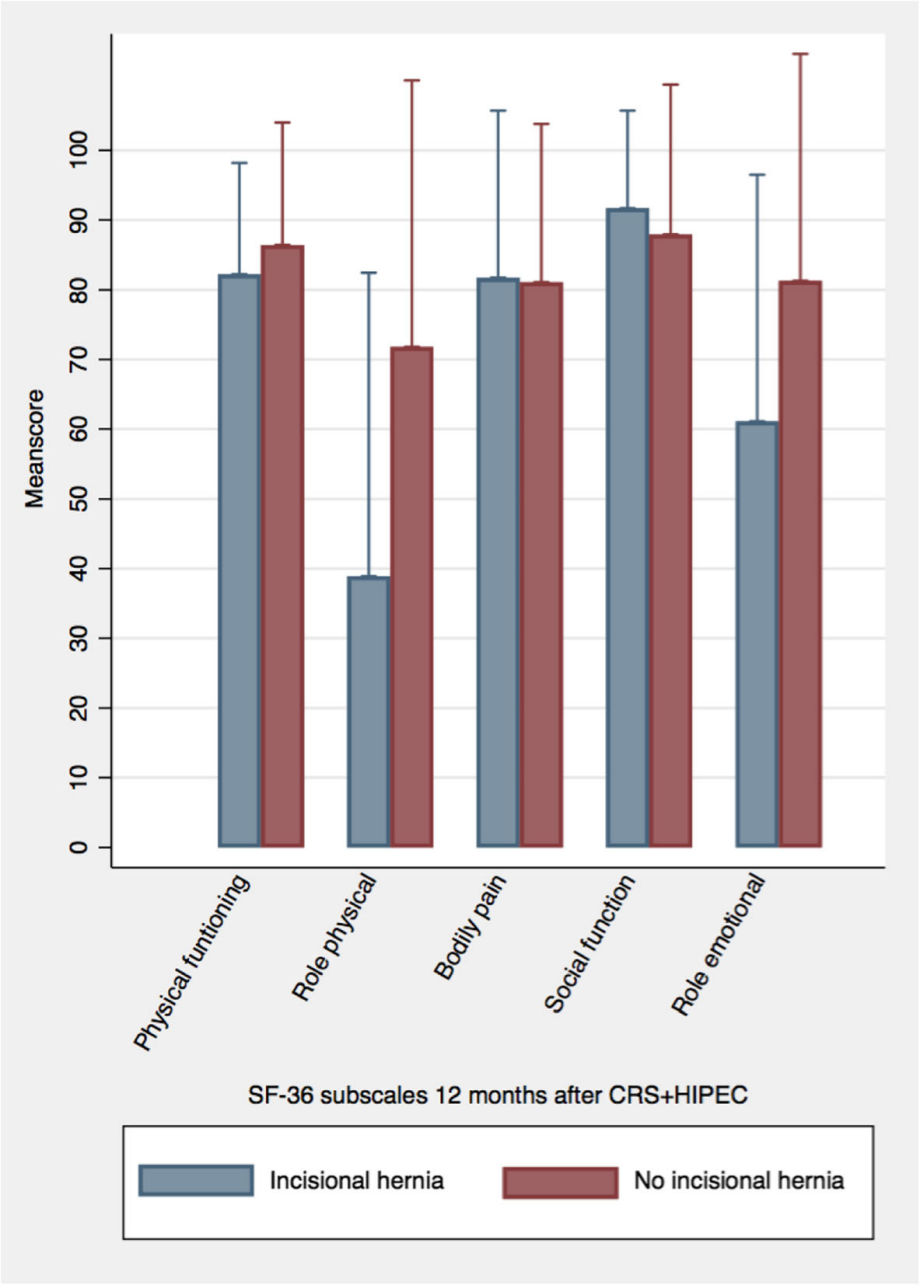
Health-related Quality of Life measured by the Short Form (SF-36). Mean scores from the Short Form (SF-36) (acute version) 12 months after cytoreductive surgery with hyperthermic intraperitoneal chemotherapy (CRS + HIPEC). The following subscales are presented: Physical functioning, Role-physical, Bodily pain, Social functioning, and Role-emotional. Presented as the mean scores with standard deviations

**Fig. 3:**
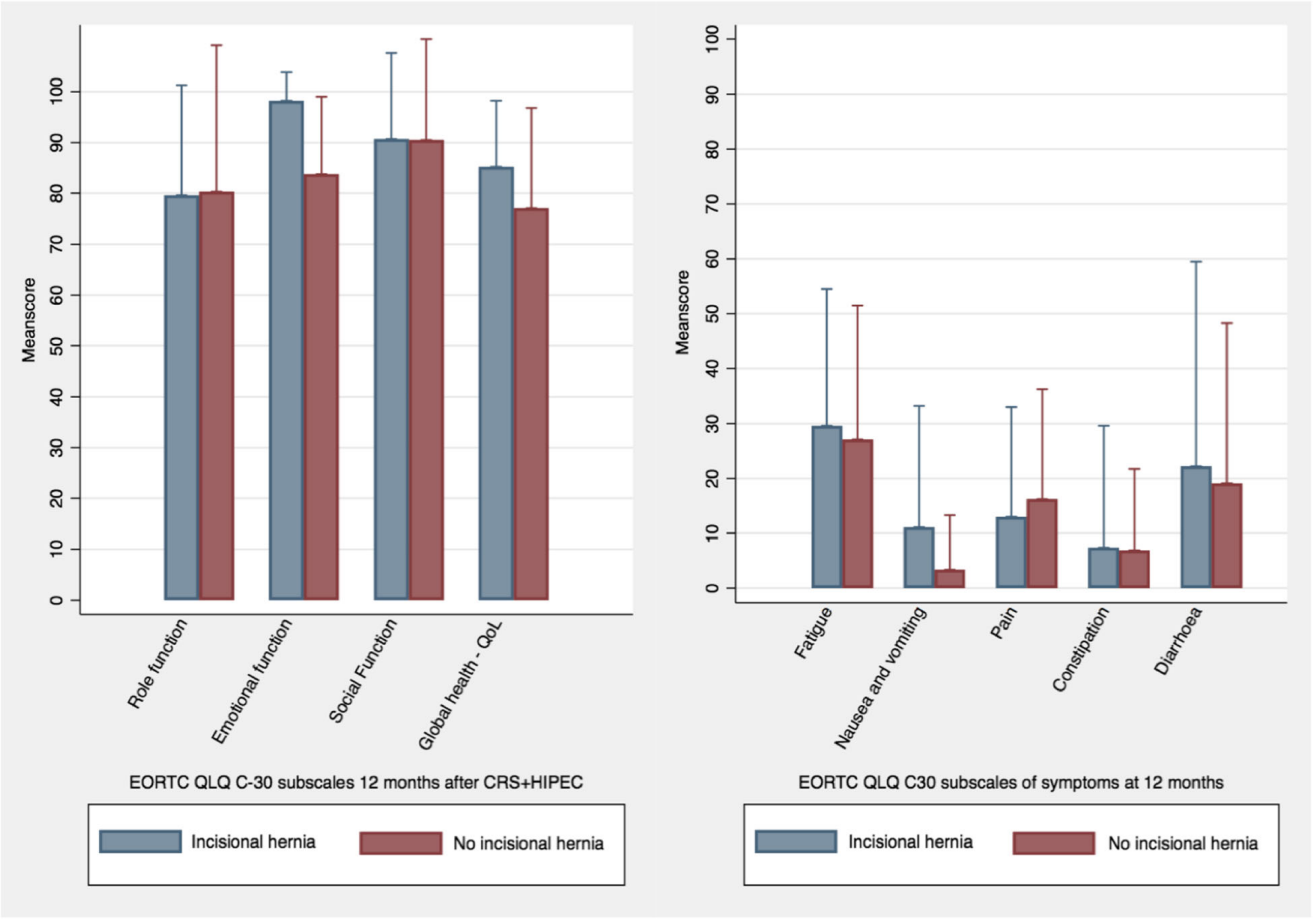
Health-related Quality of Life measured by European Organization for Research and Treatment of Cancer (EORTC) Quality of Life Core Questionnaire (QLQ-C30). Mean scores 12 months after cytoreductive surgery with hyperthermic intraperitoneal chemotherapy (CRS + HIPEC). The following subscales of symptoms are presented: Fatigue, Nausea and vomiting, Pain, Constipation, and Diarrhoea. The following subscales of function are presented: Role-physical, Emotional functioning, Social functioning, and Global health status. Presented as the mean values with standard deviations

Twelve-month scores from SF-36 and EORTC QLQ-C30 are depicted in Figs. 1 and 2. The mean scores from SF-36 and EORTC QLQ-C30 are presented in (Fig. 3) and Table 2. Comparing mean scores, SF-36 shows that +IH patients reported a statistically and clinically significant lower HRQoL with regard to *Role-physical* and a clinically significant lower level of *Role-emotional.* EORTC QLQ-C30 shows that +IH patients reported a significantly higher level of *emotional functioning*.

**Table 2:** Mean scores of scales from the Short Form (SF-36) and the European Organization for Research and Treatment of Cancer (EORTC) Quality of Life Core Questionnaire (QLQ-C30) 12 months after cytoreductive surgery with hyperthermic intraperitoneal chemotherapy (CRS + HIPEC). Presented as the mean values with standard deviations. The mean difference between patients with and without an incisional hernia is presented with 95% confidence interval

	Patients with incisional hernia (*n* = 9)	Patients without incisional hernia (*n* = 64)	Mean difference
SF-36			
Physical functioning	82.2 (16.0)	86.4 (17.6)	− 4.2 (− 16.6; 8.2)
Role-physical	38.9 (43.5)	71.8 (38.2)*	− 32.9 (− 60.6; − 5.3)
Bodily pain	81.7 (24.0)	81.1 (22.7)	0.6 (− 15.6;16.8)
Social functioning	91.7 (14.0)	87.9 (21.5)	3.8 (− 11.0; 18.5)
Role-emotional	61.1 (35.4)	81.3 (32.2)	− 20.2 (− 43.4; 3.1)
EORTC QLQ-C30			
Global health	85.2 (13.0)	77.1 (19.7)*	8.1 (− 5.5; 21.6)
Role functioning	79.6 (21.7)	80.2 (28.8)	− 0.6 (− 20.5; 19.3)
Emotional functioning	98.2 (5.6)	83.8 (15.2)*	14.4 (4.1; 24.6)
Social functioning	90.7 (16.9)	90.5 (19.8)*	0.2 (− 13.6; 14.1)
Fatigue	29.6 (24.8)	27.1 (24.4)	2.5 (− 14.8; 19.9)
Nausea and vomiting	11.1 (22.0)	3.4 (9.9)	7.7 (− 0.7; 16.2)
Pain	13.0 (20.0)	16.2 (20.1)	− 3.2 (− 17.5; 11.1)
Constipation	7.4 (22.2)	6.9 (14.9)*	0.5 (− 10.8; 11.8)
Diarrhea	22.2 (37.3)	19.1 (29.2)*	− 3.1 (− 18.3; 24.6)

*n = 63 patients

The EORTC QLQ-CR38 measurements found no difference in mean scores between the two groups (+IH/−IH) (data not shown).

## Discussion

In this study of a national cohort of 152 patients undergoing CRS + HIPEC, we found a CIP of IHs in the midline laparotomy incision of almost 6% within 1 year and 9% within 2 years. +IH patients had a higher median age, and a higher proportion developed fascial dehiscence compared to −IH patients. We thus cannot confirm the hypothesis that CRS + HIPEC is associated with an increased risk of IH compared to other open abdominal surgery.

Patients with an IH reported a significantly lower level of HRQoL regarding emotional and physical roles 12 months after their CRS + HIPEC procedure, as measured by SF-36. Data from the EORCT QLQ-C30 showed that +IH patients had a significantly higher level of HRQoL regarding emotional functioning. HRQoL data should be interpreted with caution, since it is uncertain if a lower level of HRQoL is caused by the impact of the IH or by other differences between the two groups.

A great variance in the incidence of IHs is reported in the literature, most plausibly due to data heterogeneity: diverse characteristics of patient groups, different definitions of IH, variable length of follow-up, dissimilar surgical techniques, and variations in the method of hernia diagnosis. Among the known risk factors for developing an IH are SSI, the closing technique, and the perioperative use of chemotherapy.

The association and causality between chemotherapy and IH is poorly described. Both pre- and postoperative chemotherapy is described as having a compromising effect on wound healing, thus described in retrospective studies [12, 13]. The intraoperative HIPEC administration is therefore hypothetically thought to increase the risk of IH and yet not described as an independent risk factor.

In the present study, we observed a low incidence of IH despite an intensive procedure that included HIPEC. Some arguments in favor of this might be that the same small team of dedicated and experienced consultants performed or supervised all the laparotomy incisions, including the fascial suturing. Further, none of the patients developed an SSI.

The gold standard of IH diagnosis is CT imaging with Valsalva maneuver, because a clinical examination can be problematized by obesity, abdominal pain, and previous surgery of the abdominal wall [26, 27]. The CT images in the present study were obtained primarily to detect any sign of cancer recurrence and therefore performed without the Valsalva maneuver; thus, such a CT scan has a low sensitivity to detect IH. Consequently, we choose to use the clinical examination as the method to detect an IH, potentially leading to an underestimation of the actual incidence. The indications for IH repair requires compelling causes, such as severe pain, repeating events of sub-ileus, or substantial impairment of functional level. We aimed only to find the clinically detectable and relevant incidence of IH.

Two systematic reviews find that HRQoL decreases in the period immediately after CRS + HIPEC but rises to similar or increased levels within 6 to 12 postoperative months [28, 29]. Whether this increase in the general physical and emotional level in patients after CRS + HIPEC from 6 to 12 months postoperatively is associated with an increase in physical activity and consequently an increased load on the abdominal wall is unknown. However, in our study, more than half of the patients who developed an IH did so within the first 12 postoperative months (8/14 patients (57.1%)). Further follow-up is needed to diagnose late-occurring IH. However, the possibility for a long-time follow-up regarding IH is limited, because patients for whom CRS + HIPEC is the recommended treatment for advanced cancer typically have a reduced progression-free survival time [2].

A Dutch study from 2012 reports on the incidence of IHs and their impact on HRQoL at approximately 12 months after open abdominal surgery. Patients with an IH reported significantly lower scores on the SF-36 components related to physical functioning, role-physical, and physical component [14]. This correlates well with our findings. Lower emotional and physical roles mean that patients have “accomplished less,” “cut down on time,” been “limited in kind,” and “had difficulty” with work and daily activities due to their physical and psychological health. The phrasing of the questions used to measure both these scales forced the patient to respond to any change in relation to the patient’s habitual well-being. With this in consideration, the lower level in emotional and physical role seems reliable.

Our results show a discrepancy between the patients’ emotional functioning as measured by the EORTC QLQ-C30 and that measured by the SF-36. EORTC QLQ-C30 has an oncological perspective and is especially sensitive in clinical trials of cancer therapy. This has not been used in earlier clinical trials that aimed to evaluate the impact of an IH on HRQoL, and we view its measurement of the impact of IH on HRQoL with skepticism [30]. On the other hand, SF-36 is commonly used when evaluating the impact of IH on HRQoL and much more [31], and we consider our results from this survey to be robust.

Although patients with an IH had a decreased level of HRQoL regarding emotional and physical roles, the low incidence of IH in this selected and small cohort does not support an indication for implanting a prophylactic mesh at time of CRS + HIPEC. The extensive CRS + HIPEC procedure results in a massive scar, which increases the risk of infection and complications when implanting a mesh [32]. This should especially be taken into consideration in cohorts with a reduced progression-free survival time [2].

### Strengths and limitations

An advantage of this study is the prospective registration of all data. It has a particular strength concerning HRQoL, reducing the occurrence of information bias. The study is based on a national population from a single centre where the same experienced surgeons performed all procedures and clinical examinations.

A limitation is the sparse follow-up, since a minimum of 3 years of follow-up is mandatory for determining the incidence of IH [8, 9]. Further, it is a small-scale study from a single center. The missing data due to non-responders and missing responses at 12 months may introduce the risk of selection bias, but we do not expect a skewed response rate between patients with and without IH.

## Conclusion

CRS + HIPEC do not increase the risk of IH as measured within 12 months postoperatively, contrary to expectations. However, patients with an IH report a limitation in daily activities, which can best be explained by changes in physical and psychological health. A larger cohort from multiple centres is necessary to verify our findings.

## Abbreviations

CIP: The cumulative incidence proportion
CRS: Cytoreductive surgery
EORTC QLQ-C30: The European Organization for Research and Treatment of Cancer (EORTC). Quality of Life Core Questionnaire (QLQ-C30)
EORTC QLQ-C38: The European Organization for Research and Treatment of Cancer (EORTC). Quality of Life Colorectal Questionnaire module (QLQ-CR38)
HIPEC: Hyperthermic intraperitoneal chemotherapy
HRQoL: Health-related quality of life
IH: Incisional hernia
SF-36: The Short Form
SSI: Surgical site infection
